# Number of consulting medical institutions and risk of polypharmacy in community-dwelling older people under a healthcare system with free access: a cross-sectional study in Japan

**DOI:** 10.1186/s12913-020-05205-6

**Published:** 2020-04-26

**Authors:** Toshiki Suzuki, Masao Iwagami, Shota Hamada, Tomoyuki Matsuda, Nanako Tamiya

**Affiliations:** 1 School of Medicine, University of Tsukuba, 1-1-1 Tenno-dai, Tsukuba, Ibaraki, 305-8575 Japan; 2 Department of Health Services Research, Faculty of Medicine, University of Tsukuba, 1-1-1 Tenno-dai, Tsukuba, Ibaraki, 305-8575 Japan; 3 Health Services Research and Development Center, University of Tsukuba, 1-1-1 Tenno-dai, Tsukuba, Ibaraki, 305-8575 Japan; 4 Research Department, Institute for Health Economics and Policy, Association for Health Economics Research and Social Insurance and Welfare, No.11 Toyo-Kaiji Bldg, 1-5-11 Nishi-Shimbashi, Minato-ku, Tokyo, 105-0003 Japan; 5 Department of Physical Therapy, School of Medical Health, Ibaraki Prefectural University of Health Sciences, 4669-2 Ami, Amimachi, Inashikigun, Ibaraki, 300-0394 Japan

**Keywords:** Free access, Multiple consultations, Number of consulting medical institutions, Polypharmacy

## Abstract

**Background:**

Under the Japanese free access healthcare system, patients are allowed to consult multiple medical institutions (including clinics and hospitals for general or specialist consultation) without primary care referral. This potentially increases the risk of polypharmacy. We examined the association between the number of consulting medical institutions and polypharmacy under a healthcare system with free access.

**Methods:**

Via a self-administered questionnaire, we identified people aged ≥65 years with ≥1 disease and  ≥1 consulting medical institution in a Japanese city in 2016. The exposure of interest was the number of consulting medical institutions (1, 2, or ≥3) and the outcome was polypharmacy (use of ≥6 types of drugs). We performed a multivariate logistic regression analysis, adjusting for age, sex, household economy, and the number and type of comorbidities. To minimize confounding effects, we also performed propensity-score-matched analysis, categorizing patients into two groups: 1 and  ≥2 consulting medical institutions.

**Results:**

Of 993 eligible individuals (mean (standard deviation) age: 75.1 (6.5) years, men: 52.6%), 15.7% (156/993) showed polypharmacy. Proportions of polypharmacy were 9.7% (50/516), 16.6% (55/332), and 35.2% (51/145) for people who consulted 1, 2, and  ≥3 medical institutions, respectively. Relative to people who consulted 1 medical institution, adjusted odds ratios (95% confidence intervals) for polypharmacy were 1.50 (0.94–2.37) and 3.34 (1.98–5.65) for those who consulted 2 and  ≥3 medical institutions, respectively. In propensity score matching, of 516 and 477 patients who consulted 1 and  ≥2 medical institutions, 307 pairs were generated. The proportion of polypharmacy was 10.8% (33/307) and 17.3% (53/307), respectively (*P* = 0.020). The odds ratio for polypharmacy (≥2 vs. 1 consulting medical institution) was 1.73 (95% confidence interval 1.09–2.76).

**Conclusions:**

Patients who consulted more medical institutions were more likely to show polypharmacy. The results could encourage physicians and pharmacists to collect medication information more actively and conduct appropriate medication reviews. Strengthening primary care is needed to address the polypharmacy issue, especially in countries with healthcare systems with free access.

## Background

Polypharmacy (i.e., use of multiple drugs) is known to be associated with an increased likelihood of adverse drug events, hospital admission, and mortality, particularly in older patients [1–5]. Under the public health insurance system with free access in Japan, patients choose which medical institutions they want to consult and may look up multiple medical institutions or specialists. Moreover, unlike other countries, there is no strict requirement to consult the hospitals of referral of primary care physicians. In Japan, doctors with any specialty can open a clinic for general care or specialist care in the community, and large hospitals can install both primary care and specialist care departments. Under this healthcare system, patients are allowed to visit any medical institution(s), including clinics and hospitals for general or specialist consultation. Although patients are advised to consult one physician regularly as a responsible primary care physician, a recent report suggested that the proportion of adults in Japan with a primary care physician was only 53.7% [6]. Moreover, older patients could be more likely to consult multiple medical institutions with different specialties because of multi-morbidity.

Previous studies have reported that the number and type of comorbidities experienced were associated with polypharmacy [7–10]. However, to the best of our knowledge, no studies have examined the association between the number of consulting medical institutions and the risk of polypharmacy, within the free access system, in older people living in the community. We hypothesized that consulting multiple medical institutions is an independent factor of polypharmacy and older patients who consulted multiple medical institutions are more likely to show polypharmacy, regardless of the number and type of comorbidities. Therefore, we aimed to examine an independent association between the number of consulting medical institutions and the risk of polypharmacy, using data from a cross-sectional survey conducted in a Japanese city.

## Methods

### Data source

We used data from a self-administered questionnaire survey conducted in December 2016, the original purpose of which was to formulate a welfare plan for the older citizens in Tsukuba City, Ibaraki, Japan, while the data have been secondarily used for research [11, 12]. This questionnaire was performed by postal delivery and collection. Tsukuba City had a population of approximately 220,000, with approximately 42,000 people aged ≥65 years (19.1%), in 2016. As part of the survey, the questionnaire was sent randomly to 1500 residents aged 65–74 years and 1500 people aged ≥75 years who lived at home and were not certified long-term care needs. The response rates were 50.6 and 53.2% for people aged 65–74 and  ≥75 years, respectively. Therefore, the overall response rate was 51.9% (1557/3000). In addition, we restricted the analysis to people reporting having at least one disease, who were consulting at least one medical institution regularly (*n* = 993). This was because people with no disease and those currently visiting no medical institution for routine care are not at risk for polypharmacy. All patient identifiers were removed from the dataset. The present study was approved by the ethics committee of the University of Tsukuba (#1166). Because of the anonymous nature of the data, the need for informed consent from individuals was waived.

### Independent variable, outcome, and covariates

The independent variable was the number of consulting medical institutions (including clinics and hospitals for general or specialist consultation), based on responses to the question, “How many medical institutions do you currently visit for routine care?” The outcome of interest was polypharmacy, defined as the use of at least six drugs, following a study in which the risk of adverse drug events increased significantly in hospitalized older patients using six or more drugs [13]. We identified polypharmacy based on responses to the question, “How many types of prescribed medications do you currently take?”

As potential confounders, according to a directed acyclic graph (Additional file 1), we considered age, sex, household economy, and the number and type of comorbidities [7–10]. The household economy was based on responses to the subjective self-administered question, “How do you feel about your current daily situation economically?” Possible responses were as follows: “Very poor,” “Poor,” “Normal,” “Rich,” and “Very rich.” The number and type of comorbidities were based on responses to the question, “Choose the diseases for which you are currently receiving treatment, from the following list (multiple answers possible).” The list of diseases was as follows: hypertension, stroke, heart disease, diabetes mellitus, dyslipidemia, respiratory disorder, gastrointestinal disorder, renal urologic disorder, musculoskeletal disorder, injury, malignancy, hematologic disease, depression, dementia, Parkinson’s disease, ear disorder, and other diseases.

### Statistical analysis

We described patient characteristics for 993 participants reporting having at least one disease and consulting at least one medical institution regularly. We determined the prevalence of polypharmacy according to the number of consulting medical institutions (one, two, and three or more).

We performed univariate and multivariate logistic regression analyses to estimate unadjusted and adjusted odds ratios with 95% confidence intervals (CIs) for the association between the number of consulting medical institutions and polypharmacy. To develop the multivariate logistic regression model, we selected variables significantly associated with polypharmacy in the univariate logistic regression models, at a threshold *p*-value of <0.05. As a sensitivity analysis, we added interaction terms among the comorbidities included in the multivariate logistic regression model.

Further, we performed propensity score matching as an additional analysis to better examine the independent association between the number of consulting medical institutions and polypharmacy [14]. In this analysis, we categorized the participants into two groups: those who consulted one and at least two medical institutions. The propensity score was estimated using the aforementioned covariates, and the C statistic was calculated to evaluate the goodness of fit. One-to-one nearest-neighbor matching was performed based on estimated propensity scores for each participant, using a caliper within 0.20 standard deviations of the propensity score distribution. Of the propensity-score-matched patients, we compared the proportions of those who showed polypharmacy between groups via a chi-square test, followed by univariate logistic regression analysis of the association between the number of consulting medical institutions (at least two vs. one) and polypharmacy. As a sensitivity analysis, we repeated the propensity-score-matched analysis by categorizing patients into those who had consulted two or fewer and three or more medical institutions.

The significance level was set at *p* <0.05. All statistical analyses were performed using STATA version 14.2.

## Results

**Fig. 1:**
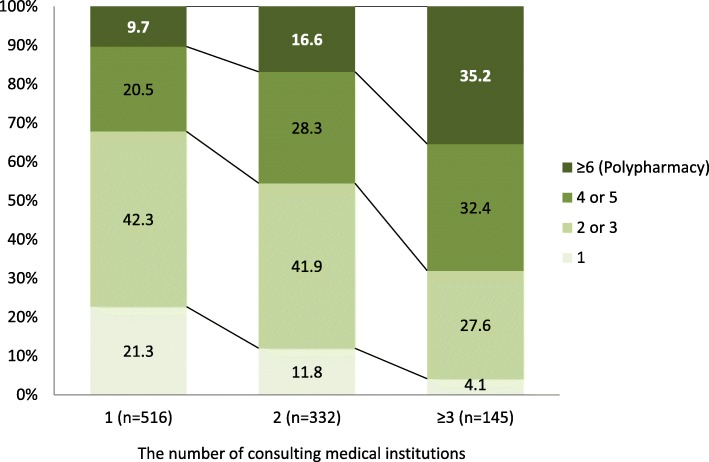
Number of prescribed drugs according to number of consulting medical institutions

Table 1 shows the baseline characteristics of the 993 eligible patients. Their mean age was 75.1 (standard deviation 6.5) years, and 52.6% were men. As shown in Fig. 1, patients who consulted higher numbers of medical institutions tended to be prescribed a higher number of drugs, relative to those who consulted fewer institutions.

**Table 1:** Characteristics of study participants

Factors	*N* = 993
	n (%)
Age (year old, mean ± SD)	75.1 ± 6.5
Sex (men)	522 (52.6)
The number of consulting medical institutions	
1	516 (52.0)
2	332 (33.4)
≥3	145 (14.6)
Household economy	
Very poor	57 (5.9)
Poor	198 (20.5)
Normal	633 (65.4)
Rich	65 (6.7)
Very rich	15 (1.6)
Not answered	25 (2.5)
Comorbidities	
Hypertension	559 (56.3)
Stroke	33 (3.3)
Heart disease	132 (13.3)
Diabetes mellitus	179 (18.0)
Dyslipidemia	157 (15.8)
Respiratory disorder	62 (6.2)
Gastrointestinal disorder	84 (8.5)
Renal urologic disorder	106 (10.7)
Musculoskeletal disorder	103 (10.4)
Injury	21 (2.1)
Malignancy	49 (4.9)
Hematologic disease	18 (1.8)
Depression	11 (1.1)
Dementia	11 (1.1)
Parkinson’s disease	1 (0.1)
Ear disorder	54 (5.4)
Other	108 (10.9)
Number of comorbidities	
1	447 (45.0)
2	325 (32.7)
3	133 (13.4)
4	67 (6.7)
≥5	21 (2.1)

*SD* standard deviation

In the univariate analysis, in comparison with participants who consulted one medical institution, the unadjusted odds ratios (95% CIs) for polypharmacy were 1.85 (1.23–2.79) and 5.06 (3.23–7.92) for those who consulted two and three or more medical institutions, respectively (Table 2). In the multivariate analysis, the association was attenuated, but an association between the number of consulting medical institutions and polypharmacy remained; adjusted odds ratios (95% CIs) were 1.50 (0.94–2.37) and 3.34 (1.98–5.65) for those who consulted two and three or more medical institutions, respectively. In the sensitivity analysis that included interaction terms among the comorbidities in the multivariate logistic regression model (i.e., heart diseases, diabetes mellitus, gastrointestinal disorders, renal urologic disorders, and musculoskeletal disorders), the adjusted odds ratios (95% CIs) were 1.57 (0.98–2.50) and 3.63 (2.11–6.24) for those who consulted two and three or more medical institutions, respectively.

**Table 2:** Results of univariate and multivariate logistic regression analyses for polypharmacy

	Unadjusted OR (95% CI)	Adjusted OR (95% CI)
The number of consulting medical institutions		
1	1 (reference)	1 (reference)
2	1.85 (1.23–2.79)	1.50 (0.94–2.37)
≥3	5.06 (3.23–7.92)	3.34 (1.98–5.65)
Age (every 1 year old increase)	1.07 (1.04–1.10)	1.07 (1.03–1.10)
Sex		
Women	1 (reference)	1 (reference)
Men	1.81 (1.27–2.59)	1.98 (1.26–3.10)
Household economy^a^		
Very poor	1.94 (1.02–3.69)	1.53 (0.72–3.26)
Poor	1.56 (1.03–2.34)	1.50 (0.95–2.38)
Normal	1 (reference)	1 (reference)
Rich^b^	0.66 (0.31–1.42)	0.73 (0.32–1.66)
Very rich^b^		
Comorbidities		
Hypertension	1.21 (0.86–1.72)	
Stroke	1.20 (0.49–2.96)	
Heart disease	3.81 (2.53–5.74)	2.89 (1.75–4.76)
Diabetes mellitus	2.49 (1.69–3.67)	2.49 (1.54–4.02)
Dyslipidemia	1.20 (0.77–1.88)	
Respiratory disorder	1.79 (0.97–3.29)	
Gastrointestinal disorder	2.35 (1.42–3.91)	1.71 (0.92–3.19)
Renal urologic disorder	2.25 (1.41–3.59)	1.04 (0.56–1.92)
Musculoskeletal disorder	2.35 (1.47–3.76)	2.44 (1.34–4.47)
Injury	2.19 (0.84–5.74)	
Malignancy	1.80 (0.92–3.54)	
Hematologic disease	1.55 (0.50–4.76)	
Depression	2.03 (0.53–7.74)	
Dementia	3.12 (0.90–10.79)	
Parkinson’s disease	Not available	
Ear disorder	1.08 (0.52–2.25)	
Other	1.08 (0.63–1.85)	
The number of comorbidities		
1	1 (reference)	1 (reference)
2	2.41 (1.51–3.85)	1.52 (0.90–2.55)
3	4.28 (2.51–7.29)	1.58 (0.83–2.99)
4	9.31 (5.09–17.04)	2.59 (1.24–5.44)
≥5	17.30 (6.78–44.09)	2.24 (0.68–7.43)

*CI* confidence interval, *OR* odds ratio

^a^25 people with missing data were not included in the multivariate logistic regression analyses

^b^People in the rich and very rich categories were grouped because of the small number of participants

**Fig. 2:**
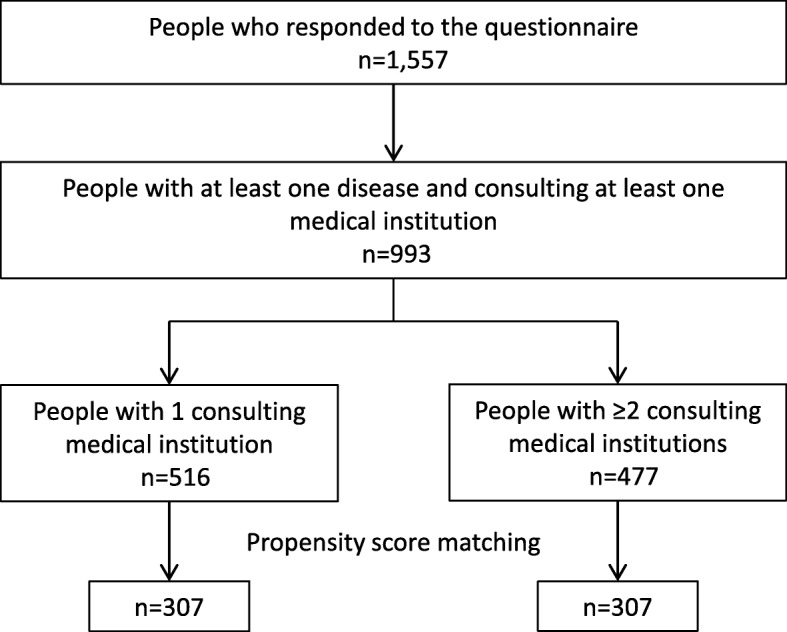
Outline of patient selection in the propensity-score-matched analysis

As shown in Fig. 2, of the 516 and 477 participants who consulted one and at least two medical institutions, 307 propensity score-matched pairs were generated, with a C statistic of 0.716. Following propensity score matching, the baseline patient characteristics were well balanced between groups (Table 3). The proportions of patients who showed polypharmacy were 10.8% (33/307) and 17.3% (53/307) in matched patients who had consulted one and at least two medical institutions (*P* = 0.020). Patients who consulted two or more medical institutions were more likely to show polypharmacy relative to matched patients who consulted one medical institution, with an odds ratio of 1.73 (95% CI, 1.09–2.76).

**Table 3:** Characteristics of participants before and after propensity score matching

	Before propensity score matching			After propensity score matching		
	1 consulting medical institution (*N* = 516)	≥2 consulting medical institutions (*N* = 477)	*P* value	1 consulting medical institution (*N* = 307)	≥2 consulting medical institutions (*N* = 307)	*P* value
Age (year old, mean ± SD)	74.5 ± 6.7	75.8 ± 6.2	0.003	74.9 ± 7.1	75.0 ± 5.9	0.956
Sex (men), n (%)	276 (53.5)	246 (51.6)	0.546	154 (50.2)	159 (51.8)	0.686
Household economy, n (%)						
Very poor	26 (5.0)	31 (6.5)	0.479	15 (4.9)	17 (5.5)	0.594
Poor	92 (17.8)	106 (22.2)		63 (20.5)	67 (21.8)	
Normal	342 (66.3)	291 (61.0)		208 (67.8)	193 (62.9)	
Rich	35 (6.8)	30 (6.3)		16 (5.2)	25 (8.1)	
Very rich	8 (1.6)	7 (1.5)		5 (1.6)	5 (1.6)	
Not answered^a^	13 (2.5)	12 (2.5)		0	0	
Comorbidities, n (%)						
Hypertension	284 (55.0)	275 (57.7)	0.407	168 (54.7)	165 (53.8)	0.808
Stroke	11 (2.1)	22 (4.6)	0.029	9 (2.9)	7 (2.3)	0.612
Heart disease	60 (11.6)	72 (15.1)	0.108	42 (13.7)	38 (12.4)	0.632
Diabetes mellitus	96 (18.6)	83 (17.4)	0.622	52 (16.9)	59 (19.2)	0.463
Dyslipidemia	84 (16.3)	73 (15.3)	0.674	51 (16.6)	47 (15.3)	0.659
Respiratory disorder	23 (4.5)	39 (8.2)	0.016	21 (6.8)	15 (4.9)	0.303
Gastrointestinal disorder	29 (5.6)	55 (11.5)	0.001	27 (8.8)	23 (7.5)	0.555
Renal urologic disorder	34 (6.6)	72 (15.1)	<0.001	28 (9.1)	29 (9.5)	0.889
Musculoskeletal disorder	34 (6.6)	69 (14.5)	<0.001	31 (10.1)	26 (8.5)	0.487
Injury	7 (1.4)	14 (2.9)	0.084	7 (2.3)	6 (2.0)	0.779
Malignancy	26 (5.0)	23 (4.8)	0.875	13 (4.2)	18 (5.9)	0.357
Hematologic disease	7 (1.4)	11 (2.3)	0.263	4 (1.3)	5 (1.6)	0.737
Depression	3 (0.6)	8 (1.7)	0.099	3 (1.0)	2 (0.7)	0.653
Dementia	6 (1.2)	5 (1.1)	0.863	4 (1.3)	2 (0.7)	0.412
Parkinson’s disease	0	1 (0.2)	0.298	0	0	
Ear disorder	24 (4.7)	30 (6.3)	0.255	14 (4.6)	11 (3.6)	0.540
Other	52 (10.1)	56 (11.7)	0.401	36 (11.7)	34 (11.1)	0.800
The number of comorbidities, n (%)						
1	300 (58.1)	147 (30.8)	<0.001	142 (46.3)	141 (45.9)	0.422
2	150 (29.1)	175 (36.7)		111 (36.2)	117 (38.1)	
3	41 (8.0)	92 (19.3)		32 (10.4)	37 (12.1)	
4	17 (3.3)	50 (10.5)		14 (4.6)	9 (2.9)	
≥5	8 (1.6)	13 (2.7)		8 (2.6)	3 (1.0)	

*SD* standard deviation

^a^25 people with missing data were not included in the propensity-score-matched analysis

In the sensitivity analysis, propensity matching was conducted for participants who consulted two or fewer and three or more medical institutions, and the resultant C statistic was 0.757 (Additional file 2 and Additional file 3). The proportions of patients who showed polypharmacy were 19.7% (26/132) and 34.4% (46/132) in matched patients who consulted two or fewer and three or more medical institutions (*P* = 0.006), with an odds ratio of 2.18 (95% CI, 1.25–3.81).

## Discussion

Free access to medical institutions is granted under the existing healthcare system in Japan; therefore, physicians do not always know which medical institutions patients have consulted or which drugs they have been prescribed, although patients are encouraged to consult specialists through referrals from primary care physicians. The current results showed that patients who consulted a high number of medical institutions tended to be at risk of polypharmacy. It is difficult for physicians to check and control the number of drugs prescribed by other physicians, particularly those at other medical institutions. The same is true of pharmacists. A likely explanation for the present results is that the number of opportunities to review and deprescribe some medications, if appropriate, was insufficient because physicians could not have known about or controlled all drugs prescribed by other physicians. In Japan, electronic medical records are available within the same institution. Thus, even if a patient consulted several specialists at the same medical institution, it would be easy for specialists at the same institution to check and control patients’ prescribed drugs. However, electronic medical records cannot be shared by different institutions, meaning that physicians at different institutions cannot see what drugs have been prescribed by other physicians in different institutions. Therefore, patients visiting several institutions are more likely to be exposed to the risk of polypharmacy than patients consulting several specialists at the same institution, even if the number of consulting physicians is the same. According to previous studies conducted in Spain, the number of different specialists to which the patients were referred by their family physicians was significantly associated with adverse drug events and the high number of prescribers was a strong predictor of polypharmacy [15, 16]. Although the healthcare system in Spain is different from Japan, these previous results are consistent with our findings.

Polypharmacy has been associated with the number and type of comorbidities [7–10]. Older patients with higher numbers of comorbidities were more likely to show polypharmacy [7, 8]. A previous study reported a strong correlation between the number of diagnosed disorders and prescribed drugs [17]. Moreover, the likelihood of the occurrence of polypharmacy has been found to differ according to the type and combination of diagnosed disorders [7, 9, 10]. In addition, previous studies have shown that disorders associated with polypharmacy included cardiovascular disorder, an endocrine disorder, gastrointestinal disorder, urologic disorder, metabolic disease, frequent urination, and insomnia [7, 9]. The results of our logistic regression analysis showed that heart disease, diabetes mellitus, gastrointestinal disorder, renal urologic disorder, and musculoskeletal disorder were associated with polypharmacy. This result was consistent with those of previous studies [7, 9]. One study demonstrated that patients with comorbidity combinations involving chronic kidney disease with osteoporosis, congestive heart failure with osteoporosis, chronic kidney disease with arthritis, and congestive heart failure with arthritis showed particularly high prevalence rates for polypharmacy [10]. Therefore, in the current study, adjustment for the number and type of comorbidities was essential to examine the independent association between polypharmacy and the number of consulting medical institutions. Our results suggest that men are more likely to show polypharmacy than women. This result might be caused by factors not included in our multivariate logistic regression analysis, such as the severity of comorbidities. A previous study conducted in Malaysia also showed that men were included in the significant risk factors [7], which is consistent with our findings. However, some studies reported that women were more likely to have polypharmacy [10, 18]. The association between sex and polypharmacy remains controversial.

The study was subject to several limitations. First, the data analyzed in the study were collected using a questionnaire in a Japanese city, and the response rate was 51.9%. Therefore, the generalizability of the results to other populations may be limited. We could not determine the characteristics of those who did not respond to the questionnaire due to the lack of relevant data. We anticipated that more health-conscious people would have been more likely to respond to the questionnaire. In our study, the proportion of polypharmacy (15.7%) was lower than in the statistics from the Ministry of Health, Labour and Welfare, in Japan. The percentages of polypharmacy (defined as using five or more prescribed drugs) among people aged 65–74 years and ≥75 years in Japan as a whole were 28.0 and 41.1%, respectively, in 2016 [19]. A previous study reported that the proportion of polypharmacy (defined as using six or more prescribed medications) among residents aged ≥65 years in an urban community in Tokyo was 28.0% [20]. The discrepancy is most likely explained by the fact that our study population consisted of healthier people without long-term care needs certification living at home. Thus, our results are not generalizable to people with long-term care needs certification. Further, the results of propensity score matching could be generalizable only to those in the range of propensity scores included in the paired analysis. Second, misclassification is likely to occur because the data were self-reported. We defined the independent variable (i.e., the number of consulting medical institutions) and outcome (i.e., polypharmacy) based on self-administered questionnaire items. If patients who consulted multiple medical institutions were more likely to overreport the number of drugs they were using, the results could have been overestimated. Third, although we adjusted for the number and type of comorbidities (i.e., important confounding factors according to previous studies [7–10]) in the multivariate logistic regression and subsequent propensity score analyses, residual confounding effects cannot be excluded entirely, as it was an observational study. For example, we were unable to obtain information regarding the severity of comorbid conditions. Patients with severe comorbidities could have been more likely to consult multiple medical institutions and receive higher numbers of prescriptions. Finally, we were unable to obtain details of prescriptions or the types of medication prescribed to patients. We could not judge the appropriateness of the drugs prescribed, because patients’ prescription content, especially that regarding potentially inappropriate medication, was not taken into consideration in the study. Some patients might have required six or more drugs because of their complex conditions (i.e., appropriate polypharmacy). Further studies are needed, in which patients’ prescription contents, especially regarding potentially inappropriate medications, are considered to evaluate the quality of polypharmacy. Despite its limitations, this study demonstrated that patients with a high number of consulting medical institutions tended to be exposed to a risk of polypharmacy.

## Conclusions

The results of the study showed that patients who consulted a higher number of medical institutions were more likely to show polypharmacy, regardless of the number or type of comorbidities, relative to those who consulted fewer institutions. The results could encourage physicians and pharmacists to collect medication information more actively and conduct appropriate medication reviews. A responsible primary care physician who coordinates and reviews patients’ medications is essential, especially for patients with multi-morbidity, under healthcare systems with free access to medical institutions, including Japan. Strengthening primary care is necessary to address the polypharmacy issue. This study could not assess the appropriateness of the prescribed drugs. However, our results could contribute to identify the actions aimed at minimizing the polypharmacy issue under healthcare systems with free access.

## Supplementary information

**Additional file 1.** Directed acyclic graph.

**Additional file 2.** Outline of patient selection in the sensitivity analysis: Propensity score matching between patients who consulted two or fewer and three or more medical institutions.

**Additional file 3.** Characteristics of patients before and after propensity score matching in the sensitivity analysis: Propensity score matching between patients who consulted two or fewer and three or more medical institutions.

## Data Availability

The data that support the findings of this study are available from the local government of Tsukuba City, Ibaraki, Japan, but restrictions apply to the availability of these data, which were used under administrative permissions for the current study, and so are not publicly available.

## Abbreviations

CI: Confidence Interval
SD: Standard Deviation
