# Supplementary material for: Number of consulting medical institutions and risk of polypharmacy in community-dwelling older people under a healthcare system with free access: a cross-sectional study in Japan
Source: BMC Health Serv Res. 2020 Apr 26;20:359. doi: 10.1186/s12913-020-05205-6 (PMC7183655; doi:10.1186/s12913-020-05205-6)
Supplement: Supplementary file 1 — Additional file 1. Directed acyclic graph. [file 12913_2020_5205_MOESM1_ESM.pptx]

## Slide 1
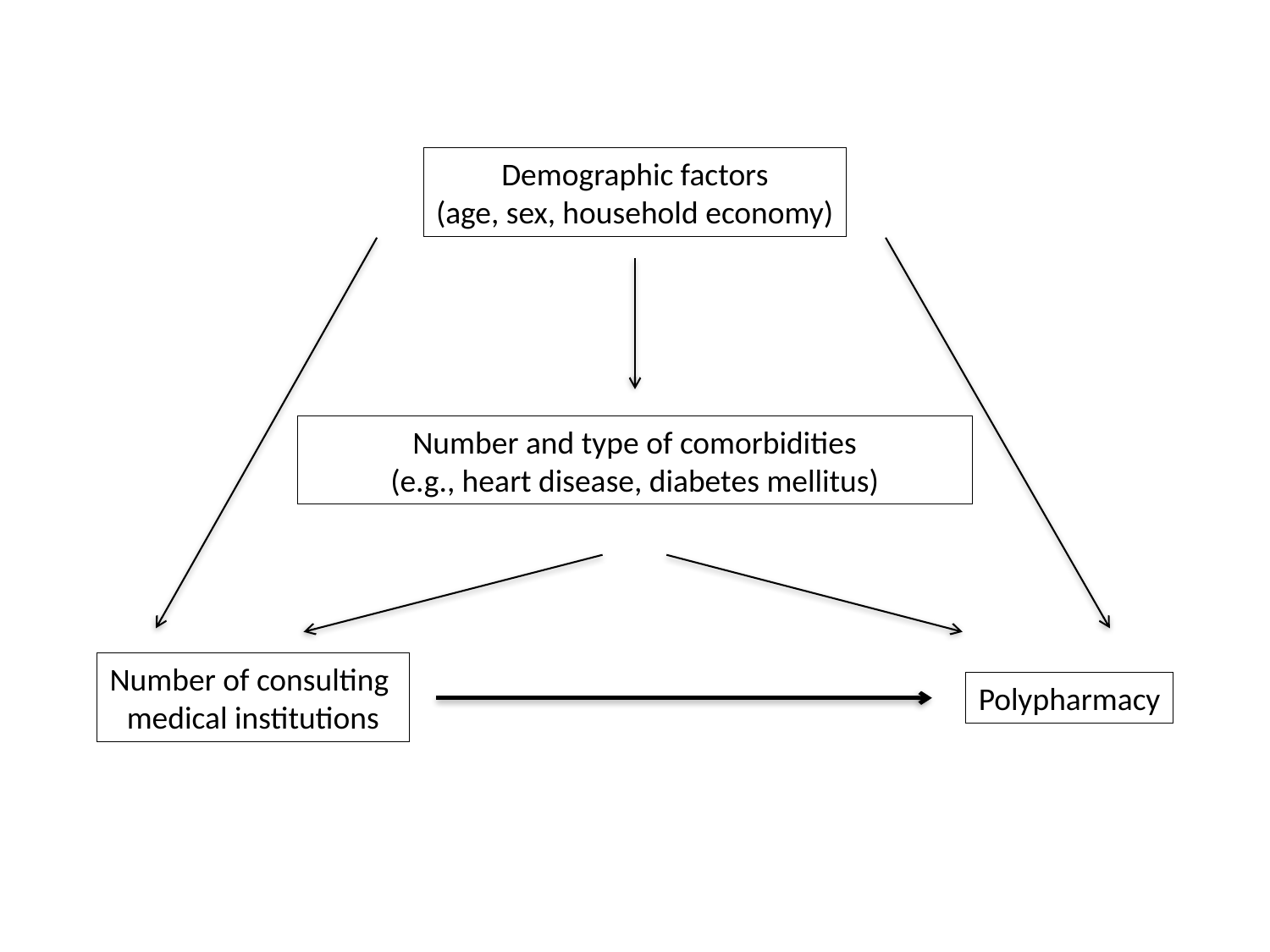

Demographic factors
(age, sex, household economy)
Number and type of comorbidities
(e.g., heart disease, diabetes mellitus)
Number of consulting
medical institutions
Polypharmacy
