# Supplementary material for: Number of consulting medical institutions and risk of polypharmacy in community-dwelling older people under a healthcare system with free access: a cross-sectional study in Japan
Source: BMC Health Serv Res. 2020 Apr 26;20:359. doi: 10.1186/s12913-020-05205-6 (PMC7183655; doi:10.1186/s12913-020-05205-6)
Supplement: Supplementary file 2 — Additional file 2. Outline of patient selection in the sensitivity analysis: Propensity score matching between patients who consulted two or fewer and three or more medical institutions. [file 12913_2020_5205_MOESM2_ESM.pptx]

## Slide 1
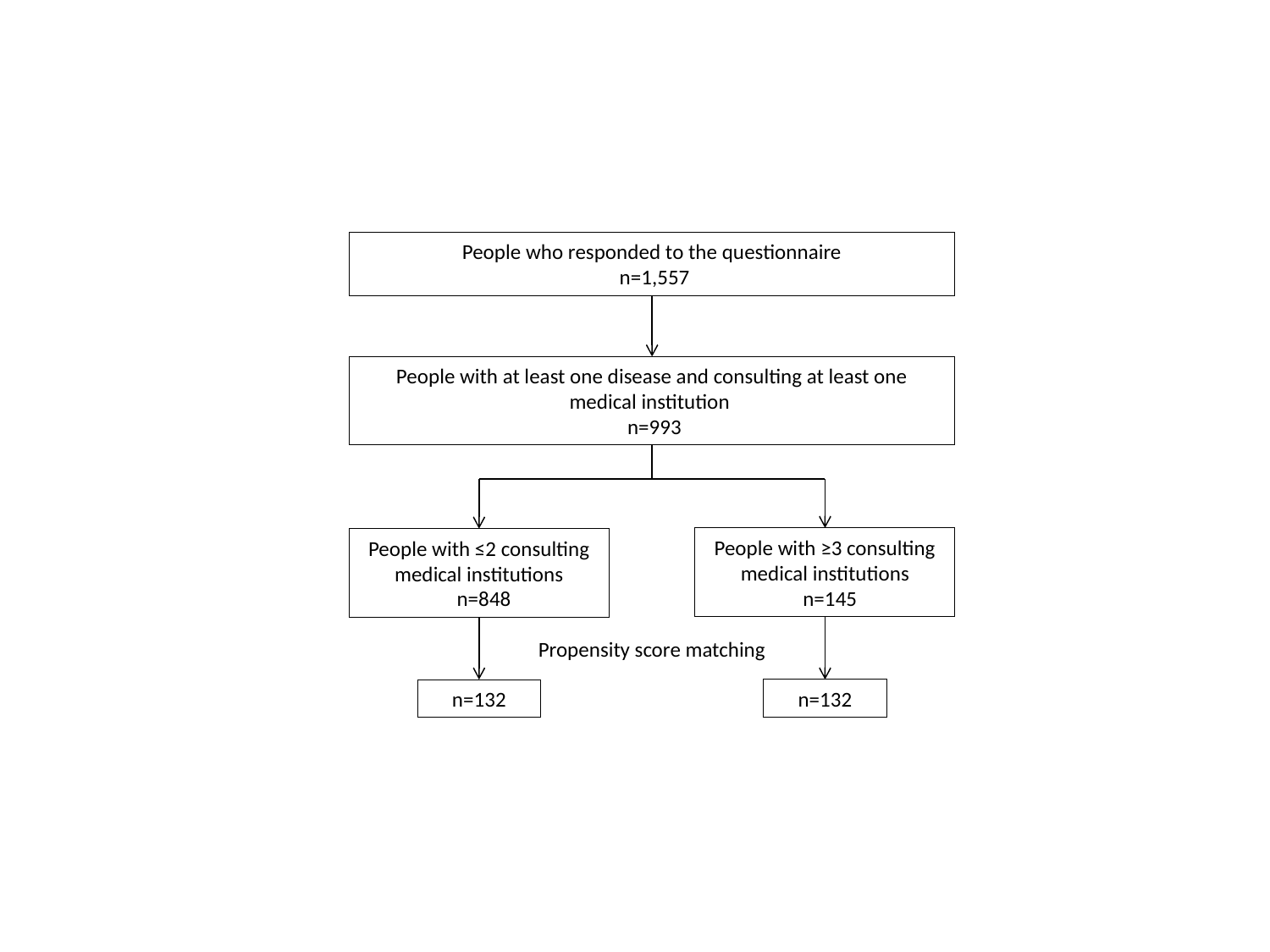

People who responded to the questionnaire
 n=1,557
People with at least one disease and consulting at least one medical institution
 n=993
People with ≥3 consulting medical institutions
 n=145
People with ≤2 consulting medical institutions
 n=848
Propensity score matching
n=132
n=132
