# Supplementary material for: Number of consulting medical institutions and risk of polypharmacy in community-dwelling older people under a healthcare system with free access: a cross-sectional study in Japan
Source: BMC Health Serv Res. 2020 Apr 26;20:359. doi: 10.1186/s12913-020-05205-6 (PMC7183655; doi:10.1186/s12913-020-05205-6)
Supplement: Supplementary file 3 — Additional file 3. Characteristics of patients before and after propensity score matching in the sensitivity analysis: Propensity score matching between patients who consulted two or fewer and three or more medical institutions. [file 12913_2020_5205_MOESM3_ESM.docx]

Additional file 3. Characteristics of participants before and after propensity score matching in sensitivity analysis: Propensity score matching between patients who consulted two or fewer and three or more medical institutions.

|  | Before propensity score matching | | | After propensity score matching | | |
| --- | --- | --- | --- | --- | --- | --- |
|  | ≤2 consulting  medical institutions  (N = 848) | ≥3 consulting  medical institutions  (N = 145) | P value | ≤2 consulting  medical institutions  (N = 132) | ≥3 consulting  medical institutions  (N = 132) | P value |
| Age (year old, mean ± SD) | 74.9 ± 6.5 | 76.3 ± 6.4 | 0.018 | 76.8 ± 7.1 | 76.1 ± 6.4 | 0.409 |
| Sex (men), n (%) | 449 (53.0) | 73 (50.3) | 0.562 | 65 (49.2) | 66 (50.0) | 0.902 |
| House economy, n (%) |  |  |  |  |  |  |
| Very poor | 46 (5.4) | 11 (7.6) | 0.639 | 9 (6.8) | 10 (7.6) | 0.793 |
| Poor | 165 (19.5) | 33 (22.8) |  | 28 (21.2) | 31 (23.5) |  |
| Normal | 544 (64.2) | 89 (61.4) |  | 85 (64.4) | 84 (63.6) |  |
| Rich | 59 (7.0) | 6 (4.1) |  | 9 (6.8) | 5 (3.8) |  |
| Very rich | 13 (1.5) | 2 (1.4) |  | 1 (0.8) | 2 (1.5) |  |
| Not answered^a^ | 21 (2.5) | 4 (2.8) |  | 0 | 0 |  |
| Comorbidities, n (%) |  |  |  |  |  |  |
| Hypertension | 476 (56.1) | 83 (57.2) | 0.803 | 81 (61.4) | 78 (59.1) | 0.706 |
| Stroke | 25 (3.0) | 8 (5.5) | 0.111 | 9 (6.8) | 8 (6.1) | 0.802 |
| Heart disease | 107 (12.6) | 25 (17.2) | 0.130 | 23 (17.4) | 24 (18.2) | 0.872 |
| Diabetes mellitus | 146 (17.2) | 33 (22.8) | 0.109 | 24 (18.2) | 31 (23.5) | 0.289 |
| Dyslipidemia | 132 (15.6) | 25 (17.2) | 0.609 | 19 (14.4) | 22 (16.7) | 0.610 |
| Respiratory disorder | 51 (6.0) | 11 (7.6) | 0.470 | 9 (6.8) | 10 (7.6) | 0.812 |
| Gastrointestinal disorder | 63 (7.4) | 21 (14.5) | 0.005 | 18 (13.6) | 16 (12.1) | 0.713 |
| Renal urologic disorder | 80 (9.4) | 26 (17.9) | 0.002 | 22 (16.7) | 21 (15.9) | 0.868 |
| Musculoskeletal disorder | 76 (9.0) | 27 (18.6) | <0.001 | 26 (19.7) | 22 (16.7) | 0.523 |
| Injury | 15 (1.8) | 6 (4.1) | 0.067 | 7 (5.3) | 6 (4.6) | 0.776 |
| Malignancy | 38 (4.5) | 11 (7.6) | 0.111 | 11 (8.3) | 8 (6.1) | 0.475 |
| Hematologic disease | 13 (1.5) | 5 (3.5) | 0.110 | 5 (3.8) | 4 (3.0) | 0.734 |
| Depression | 7 (0.8) | 4 (2.8) | 0.040 | 2 (1.5) | 3 (2.3) | 0.652 |
| Dementia | 8 (0.9) | 3 (2.1) | 0.231 | 3 (2.3) | 3 (2.3) | 1.000 |
| Parkinson's disease | 1 (0.1) | 0 | 0.679 | 0 | 0 |  |
| Ear disorder | 37 (4.4) | 17 (11.7) | <0.001 | 18 (13.6) | 15 (11.4) | 0.577 |
| Other | 90 (10.6) | 18 (12.4) | 0.520 | 16 (12.1) | 16 (12.1) | 1.000 |
| The number of comorbidities, n (%) |  |  |  |  |  |  |
| 1 | 420 (49.5) | 27 (18.6) | <0.001 | 28 (21.2) | 25 (18.9) | 0.488 |
| 2 | 282 (33.3) | 43 (29.7) |  | 44 (33.3) | 41 (31.1) |  |
| 3 | 92 (10.9) | 41 (28.3) |  | 29 (22.0) | 41 (31.1) |  |
| 4 | 42 (5.0) | 25 (17.2) |  | 21 (15.9) | 19 (14.4) |  |
| ≥5 | 12 (1.4) | 9 (6.2) |  | 10 (7.6) | 6 (4.6) |  |

SD = standard deviation.

^a^25 people with missing data were not included in the propensity-score-matched analysis.
